# Genetically induced oxidative stress in mice causes thrombocytosis, splenomegaly and placental angiodysplasia that leads to recurrent abortion

**DOI:** 10.1016/j.redox.2014.05.001

**Published:** 2014-05-14

**Authors:** Takamasa Ishii, Masaki Miyazawa, Yumi Takanashi, Maya Tanigawa, Kayo Yasuda, Hiromi Onouchi, Noboru Kawabe, Junji Mitsushita, Phil S. Hartman, Naoaki Ishii

**Affiliations:** a Department of Molecular Life Science, Tokai University School of Medicine, 143 Shimokasuya, Isehara, Kanagawa 259-1193, Japan; b Education and Research Support Center, Tokai University, 143 Shimokasuya, Isehara, Kanagawa 259-1193, Japan; c Department of Ophthalmology, Tokai University School of Medicine, 143 Shimokasuya, Isehara, Kanagawa 259-1193, Japan; d Department of Obstetrics and Gynecology, Saitama Medical Center, Jichi Medical University, 1-847 Amanuma-cho, Omiya, Saitama 330-8503, Japan; e Department of Biology, Texas Christian University, Fort Worth, TX 76129, USA

**Keywords:** Mitochondria, SDHC, Infertility, Abortion, Oxidative stress, ROS, reactive oxygen species, SDH, succinate dehydrogenase, SDHC, succinate dehydrogenase C subunit, TUNEL, terminal deoxynucleotidyl transferase dUTP nick end labeling, NGF, non-growing follicles, VEGF, vascular endothelial growth factors, VEGFR-1, VEGF receptor-1, PLGF, placenta growth factor

## Abstract

Historical data in the 1950s suggests that 7%, 11%, 33%, and 87% of couples were infertile by ages 30, 35, 40 and 45, respectively. Up to 22.3% of infertile couples have unexplained infertility. Oxidative stress is associated with male and female infertility. However, there is insufficient evidence relating to the influence of oxidative stress on the maintenance of a viable pregnancy, including pregnancy complications and fetal development. Recently, we have established *Tet-mev-1* conditional transgenic mice, which can express the doxycycline-induced mutant SDHC^V69E^ transgene and experience mitochondrial respiratory chain dysfunction leading to intracellular oxidative stress. In this report, we demonstrate that this kind of abnormal mitochondrial respiratory chain-induced chronic oxidative stress affects fertility, pregnancy and delivery rates as well as causes recurrent abortions, occasionally resulting in maternal death. Despite this, spermatogenesis and early embryogenesis are completely normal, indicating the mutation's effects to be rather subtle. Female *Tet-mev-1* mice exhibit thrombocytosis and splenomegaly in both non-pregnant and pregnant mice as well as placental angiodysplasia with reduced Flt-1 protein leading to hypoxic conditions, which could contribute to placental inflammation and fetal abnormal angiogenesis. Collectively these data strongly suggest that chronic oxidative stress caused by mitochondrial mutations provokes spontaneous abortions and recurrent miscarriage resulting in age-related female infertility.

## Highlights

• SDHC mutation induces oxidative stress in the female reproductive organs.
• Early development is completely normal in *Tet-mev-1* mice.
• Non-pregnant and pregnant *Tet-mev-1* mice exhibit thrombocytosis and splenomegaly.
• Pregnant mice have placental angiodysplasia with decreased Flt-1/ VEGFR-1.
• Habitual abortion is frequently caused with occasional maternal death in *Tet-mev-1* mice.

## Graphical abstract

**Figure:**
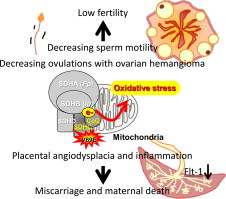

## Introduction

Human historical data in the 1950s suggests that 7%, 11%, 33%, and 87% of couples were infertile by ages 30, 35, 40 and 45, respectively [1]. Under natural conditions, 75% of 30-year old women attempting to conceive will have a conception ending in a live birth within 1 year, 66% at age 35 years, and only 44% at age 40 [2]. Spontaneous abortion refers to the unintentional termination of a pregnancy before fetal viability at 20 weeks of gestation or when fetal weight is <500 g. The incidence of such abortions is 12–24% of all pregnancies [3]. The risk of a spontaneous abortion was 8.9% in women aged 20–24 years and 74.7% in those aged 45 years or older [4]. Unexplained infertility is defined as the inability to conceive after 12 months of unprotected intercourse in couples where known causes of infertility have been ruled out. Up to 22.3% of infertile couples have unexplained infertility [5].

Oxidative stress is associated with male and female infertility [6,7]. Evidence exists supporting the role of oxidative stress in male infertility, including decreased sperm motility, sperm number, and sperm–ovum fertilization [6,8]. However, there is insufficient evidence relating to the influence of oxidative stress for maintenance of a viable pregnancy, including pregnancy complications and fetal development. Oxidative stress occurs when the generation of reactive oxygen species (ROS) and other radical species exceeds the scavenging capacity by antioxidants due to excessive production of ROS and inadequate intake or increased utilization of antioxidants. Most ROS are formed as a consequence of the mitochondrial respiratory chain, but can also be formed by exogenous exposures such as smoke and environmental pollutants [6]. It has been reported that mitochondrial mutations leading to ROS production and excessive apoptosis in a mouse model results in male infertility, but not female infertility [9].

The *mev-1* mutant of the nematode *Caenorhabditis elegans* was isolated based upon its hypersensitivity to the ROS-generating chemical methyl viologen [10]. In addition to its hypersensitivity to oxidative stress, short-lived *mev-1* mutants age precociously under hyperoxia [11–13]. The *mev-1*(*kn-1*) mutation, which results in an amino acid substitution at the 71st position from glycine to glutamate (G71E), has been identified as residing in the putative gene *cyt-1* (a human *SDHC* gene homologue), which is homologous to the succinate dehydrogenase (SDH) cytochrome *b* large subunit in complex II [14]. The biochemical pathologies of *mev-1* include elevated ROS caused by the compromised complex II resulting in electron leakage from the electron transport system [15]. These *mev-1* mutant hermaphrodites have low numbers of progeny and excessive apoptosis during fetal development [10,16].

A *mev-1*-mimic transgenic mouse was created that overexpressed the mutated SDHC^V69E^ transgene [17]. The mutated SDHC^V69E^ transgene results in the substitution of valine for glutamic acid at position 69 (V69E), which is equivalent to the *kn-1* mutation of *C. elegans* *mev-1* mutant [18,19]. This mouse had increased O_2_^•-^ levels in the mitochondria of its various tissues as well as decreased body weight and locomotion. Unfortunately, this *mev-1* transgenic mouse was infertile, which prevented propagation of the strain for further studies. Recently, we established *mev-1*-mimic (*Tet-mev-1*) conditional transgenic mice with the SDHC^V69E^ transgene using our modified Tet-On/Off construct, so that the induced SDHC^V69E^ was competitively expressed to the endogenous SDHC protein level [20]. Induction of the transgene with doxycycline led to increased mitochondrial oxidative stress that triggered excessive apoptosis, reductions in birth weight and significant delays in development in *Tet-mev-1* mice, just as in the *C. elegans* *mev-1* mutant [20].

In this report, we assessed the effects of the mitochondrial oxidative stress caused by the SDHC mutation on the male and female fertilities in mice. Specifically, we demonstrate that *Tet-mev-1* mice exhibit the same oxidative stress-induced short-lived and low fertility phenotypes that characterize the nematode mutant. Thus, this mouse model can provide a powerful opportunity to study the effects of chronic oxidative stress on infertility and abortion with age as in humans.

## Results

### Function of testis and spermatogenesis

**Fig. 1:**
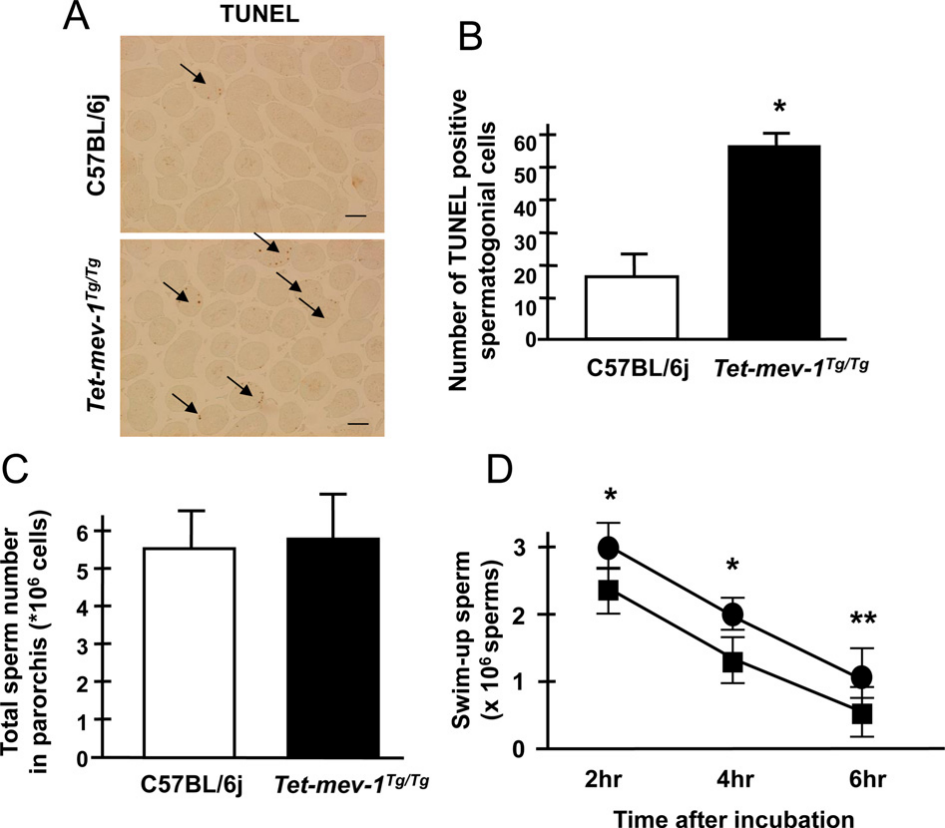
Testicle conditions, spermatogenesis and sperm motilities. (A) Cross sections of testis, showing TUNEL-stained cells (brown), which are undergoing apoptosis. Scale bar = 100 µm. (B) Statistical analysis of apoptosis in testis, using data such as portrayed in (A). Results are expressed as mean ± SD; ^*^P < 0.001; *n* = 4 in each group. (C) The chart indicates the total sperm number in parorchis. Results are expressed as mean ± SD; *n* = 7 in each group. (D) The line chart indicates the sperm motility that was measured as the swim-up sperm number after 2, 4 and 6 h incubation in HTF medium. Circles and squares indicate wild-type C57BL/6j and *Tet-mev-1* mice, respectively. Results are expressed as mean ± SD; ^*^P < 0.01, ^**^P < 0.05; *n* = 7 in each group.

Oxidative stress is well known to affect germ-cell development and testes in male infertility [21,22]. We addressed this using *Tet-mev-1*, a mouse model characterized by chronic oxidative stress owing to the doxycycline-inducible expression of a defective complex II protein (SDHC^V69E^) involved in electron transport. SDHC protein levels were first measured in testes and female reproductive organs (ovaries and uteri) of doxycycline-treated *Tet-mev-1* and wild-type C57BL/6j mice. Under these conditions, SDHC protein levels in *Tet-mev-1* mice were increased from levels equal to wild-type C57BL/6j in un-induced mice to approximately twice that of wild-type with doxycycline induction. This is consistent with our previously reported measurements in other tissues [20,23]. Next, biochemical analyses were performed in the testes of doxycycline-treated *Tet-mev-1* and wild-type C57BL/6j mice, with no detectable changes in oxidative stress and malonate-dependent cytochrome *c* oxidoreductase activity. In contrast, and unlike other tissues examined in *Tet-mev-1* mice, succinate-cytochrome *c* oxidoreductase activity, from complex II and complex III activity was essentially similar to wild-type. The energy metabolism in testes as a whole results from many of the testicular cells such as spermatocytes, spermatids and spermatozoa, which are in various stages of meiosis. These cells are powered primarily by glycolysis rather than electron transport [24]. This leads to the suggestion that a mutation affecting electron transport, such as one in complex II SDHC mutation, should have modest effects on testicle functions. It has been reported that so-called mito-mice, which harbor mitochondrial DNA-deletions that severely reduce mitochondrial functions, suffer male infertility, abnormal spermatogenesis and decreasing sperm motility [9]. We therefore examined *Tet-mev-1* mice, which also suffer chronically elevated levels of superoxide anion at a more modest rate than mito-mice, to see whether they have similar phenotypes. We observed excessive apoptosis in the spermatogonial cells of *Tet-mev-1*mice as revealed by TUNEL (terminal deoxynucleotidyl transferase dUTP nick end labeling) staining (Fig. 1A and B). However, the number of spermatozoa in *Tet-mev-1* parorchis was experimentally identical to that of wild-type C57BL/6j mice, and there were no detectible morphological abnormalities (Fig. 1C). However, spermatozoon motility was slightly decreased in *Tet-mev-1*mice compared to wild-type C57BL/6j mice (Fig. 1D).

### Function of ovaries, oogenesis, *in vitro* fertilization ability and early embryogenesis

**Fig. 2:**
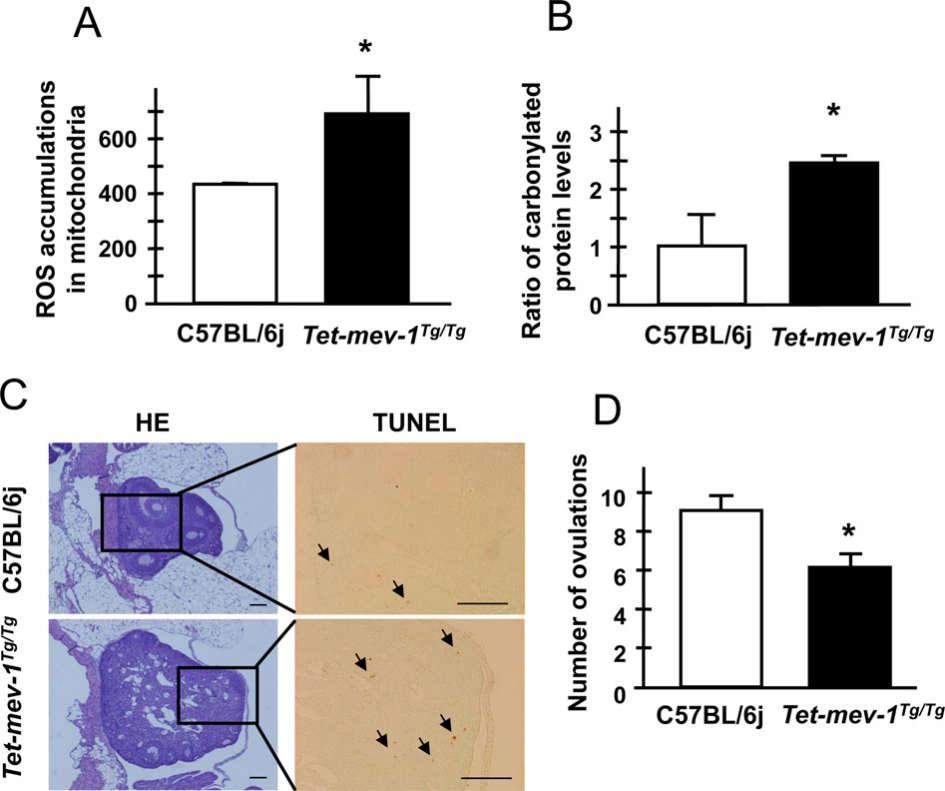
Ovary conditions and number of ovulations. (A) ROS accumulation levels in mitochondria from ovaries taken from *Tet-mev-1* or wild-type C75BL/6j mice. Results are expressed as mean ± SD; ^*^P < 0.01; *n* = 4 in each group. (B) The carbonylated protein levels in the membrane fraction protein lysate from ovaries taken from *Tet-mev-1* or wild-type C75BL/6j mice. Results are expressed as mean ± SD as the ratio compared to the levels of wild-type C75BL/6j mice under the calibration curve method with standard oxidized protein; ^*^P < 0.01; *n* = 4 in each group. (C) Micrographs of hematoxylin-eosin- and TUNEL-stained ovaries. The blocked areas feature TUNEL-positive cells that are undergoing apoptosis. Scale bar = 100 µm. (D) The number of ovulations in *Tet-mev-1* or wild-type C75BL/6j mice. Results are expressed as mean ± SD; ^*^P < 0.001; *n* = 4 in each group.

Unlike in the testes, mitochondrial ROS production and carbonylated protein levels were significantly increased in the ovaries of *Tet-mev-1* mice compared to wild-type C57BL/6j mice (Fig. 2A and B). Thus, *Tet-mev-1* mice show excessive intracellular oxidative stress, presumably because they are chronically subject to elevated levels of superoxide anion from mitochondrial complex II owing to the SDHC^V69E^ mutation. Given this, it is not surprising that the *Tet-mev-1* ovaries were swollen and vacuolated with increased angiogenesis such as ovarian hemangiomas [25], as well as increased apoptosis detected as TUNEL-positive cells in ovarian interstitial cells (Fig. 2C). In addition, the follicle maturations were not synchronously developed in *Tet-mev-1*mice (Fig. 2C). Moreover, the number of ovulations was significantly decreased in *Tet-mev-1*mice (Fig. 2D). These results are consistent with the fact that the number of non-growing follicles (NGF) declines with age as in humans. It is estimated that only 12% of the maximum pre-birth NGF population is present in 30-year old women and by the age of 40 years only 3% remain [26].

**Fig. 3:**
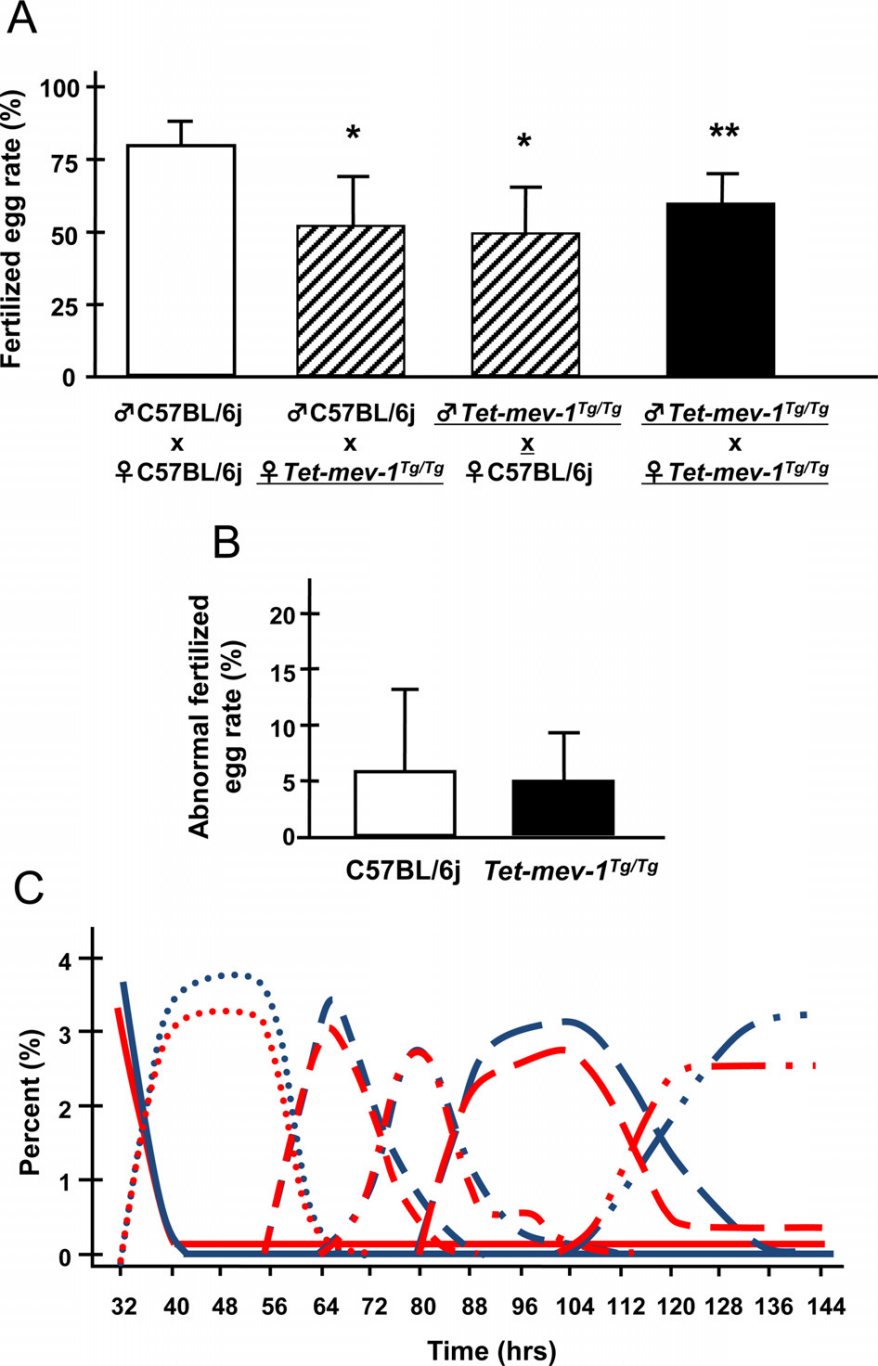
Fertility and early embryogenesis *in vitro* fertilization assay. (A) The chart indicates the fertilized egg rates using eggs and sperm from the indicated strain. Results are expressed as mean ± SD; ^*^P < 0.01; ^**^P < 0.05; *n* = 5 in each group. (B) Rates of abnormal fertilization. Results are expressed as mean ± SD; *n* = 100 in each group. (C) The progress of early embryogenesis through the blastocyst stage in *in vitro* fertilized animals. Blue or red lines indicate the developmental stage rates of C57BL/6j or *Tet-mev-1* mice. Solid lines, 1-cell embryo stage; dotted lines, 2-cell embryo stage; dashed lines, 3–4-cell embryo stage; 1-dot dashed lines, 5–8-cell embryo stage; long dashed lines, morula stage; 2-dot long dashed lines, blastocyst stage. *n* = 214 in wild-type C57BL/6j and *Tet-mev-1* mice group.

An *in vitro* fertilization assay revealed that egg fertilization was significantly decreased when using either *Tet-mev-1* sperm or eggs or both (Fig. 3A). In contrast, the percentage of abnormally fertilized eggs, *e.g*., polyspermy eggs and developmental arrested eggs before 2-cell embryo, was normal in *Tet-mev-1*mice compared to wild-type C57BL/6j mice (Fig. 3B). Similarly, early embryogenesis through the blastocyst stage was no different between *Tet-mev-1* and wild type mice (Fig. 3C). This suggests that the mitochondrial complex II SDHC^V69E^ mutation did not affect fertilization and early embryogenesis but rather exerted its effects later in development. This is logical given that energy metabolism is mainly from glycolysis in the enclosed environment during the blastomere stage [27].

### Decreasing pregnancy and delivery rates with thrombocytosis and splenomegaly

**Fig. 4:**
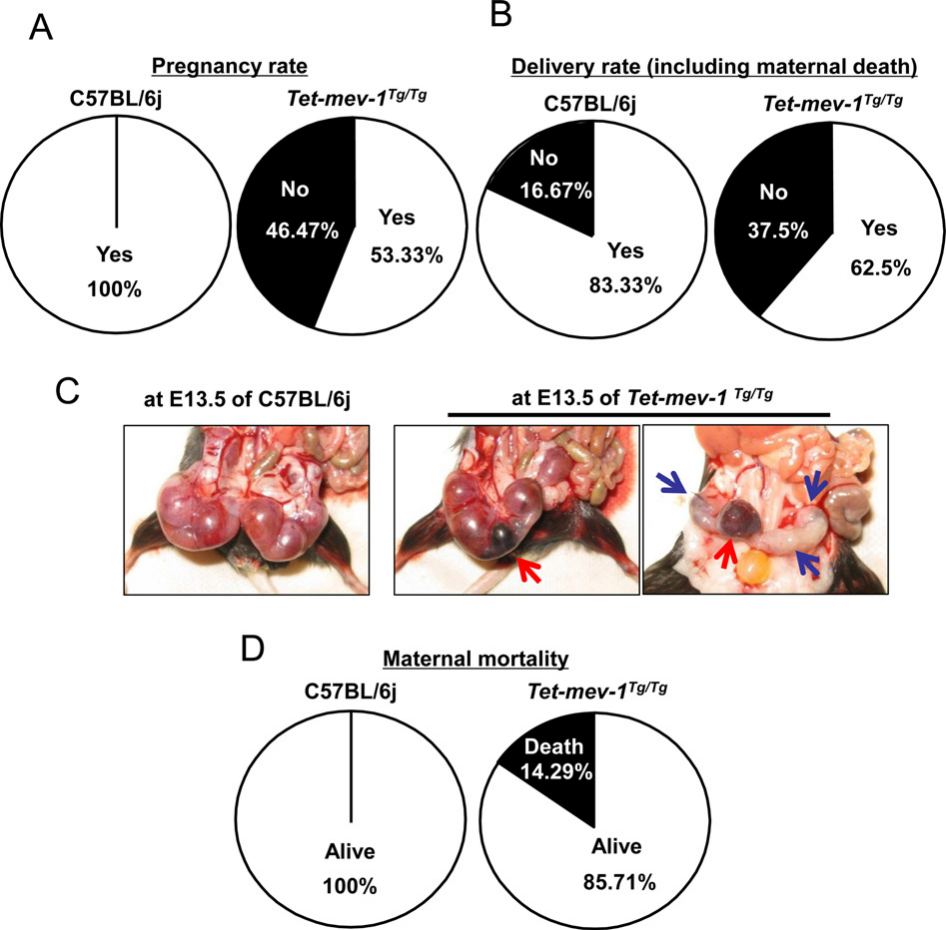
Pregnancy and delivery rates, intrauterine conditions and maternal mortality at first delivery. (A and B) Pregnancy (A) and delivery (B) rates after natural mating in each strain. The comparisons of rates between C57BL/6j and *Tet-mev-1* mice; *P* < 0.05; *n* = 15 in each group. (C) Uterine conditions at 13.5-day pregnant mice. The red and blue allows indicate the placental inflammation and fetal loss, respectively. (D) Maternal mortality at first delivery in each strain. The comparison of maternal mortality at first delivery between C57BL/6j and *Tet-mev-1* mice; *P* < 0.05; *n* = 30 in each group.

**Fig. 5:**
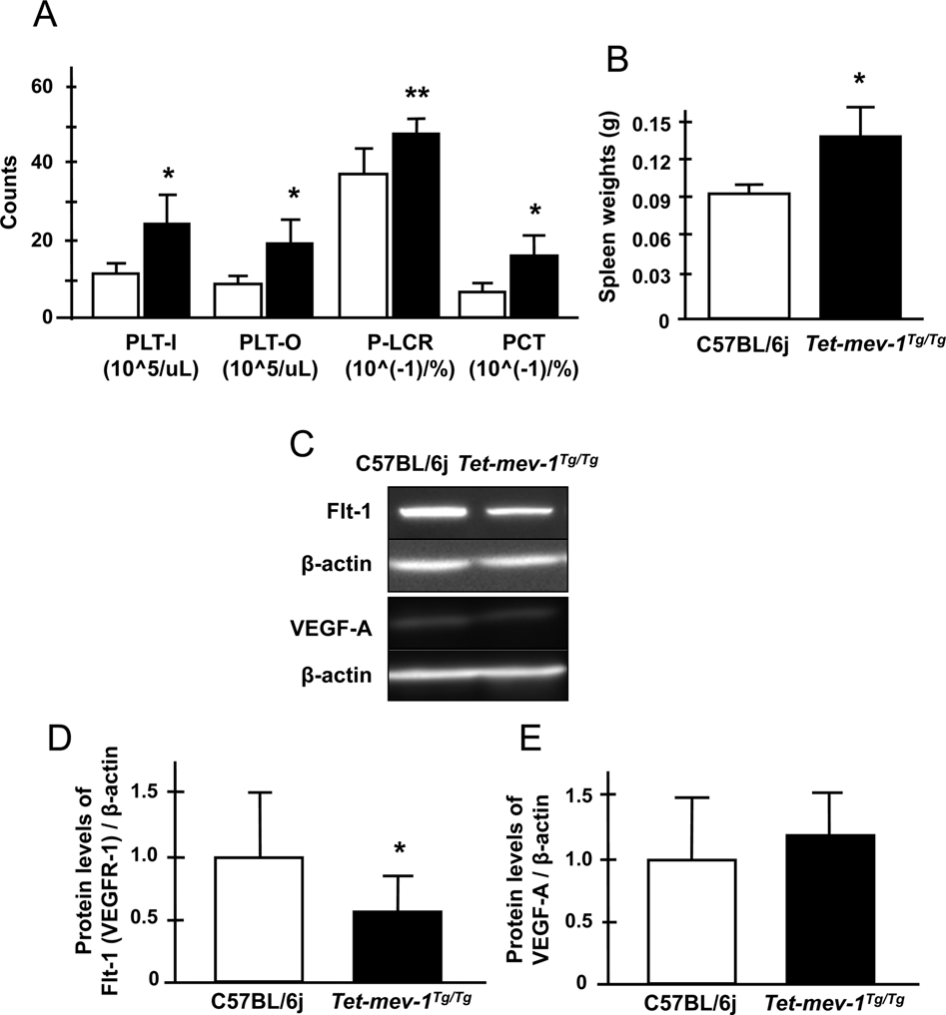
Conditions of peripheral blood and spleen weight in non-pregnant mice and condition of normal morphological placentas at 13.5-day pregnant mice. (A) The number of platelets (PLT-I indicates the impedance method used to estimate platelet counts and PLT-O indicates the optical method used to measure platelet counts.), the ratio of large platelet (P-LCR) and platelet density (PCT) derived from peripheral blood taken from non-pregnant mice using a Sysmex XT-2000iV hematology analyzer. White and black bars indicate the wild-type C57BL/6j and *Tet-mev-1* mice. Results are expressed as mean ± SD; ^*^P < 0.01; ^**^P < 0.05; *n* = 5 in each group. (B) The chart indicates the spleen weight. Results are expressed as mean ± SD; *P < 0.01; *n* = 5 in each group. (C) The results of western blot analysis using anti-Flt-1 (VEGFR-1), VEGF-A, and ß-actin antibodies. (D and E) Quantification values of Flt-1 (D) and VEGF-A (*E*) protein levels derived from western blots. Results are expressed as mean ± SD; ^*^P = 0.01; *n* = 8 in each group.

The pregnancy rate before first experience of safe delivery (primiparity) was decreased by about 50% in *Tet-mev-1*mice allowed to mate and conceive naturally (Fig. 4A). Similarly, the delivery rate was also significantly decreased in pregnant *Tet-mev-1*mice (Fig. 4B). These results indicate that the *C. elegans* short-lived *mev-1* mutant-mimics *Tet-mev-1* mice's accelerated age-related infertility. It is also the same as conditions of human age-related pregnancy rate decline. Given this, it was expected that the *Tet-mev-1*mice could demonstrate a similar unexplained age-related female infertility. Indeed, fetal loss and placental inflammation were frequently observed in 13.5-day pregnant *Tet-mev-1* mice (Fig. 4C). In humans, spontaneous abortion refers to the unintentional termination of a pregnancy before fetal viability at 20 weeks of gestation or when fetal weight is <500 g. The incidence of abortion is 12–24% of all pregnancies [3]. The risk of a spontaneous abortion was 8.9% in women aged 20–24 years and 74.7% in those aged 45 years or older [4]. Similarly, it is not surprising that the incidence of maternal death was increased at first delivery in *Tet-mev-1* mice (Fig. 4D), suggesting that reduced blood flow to the placenta contributed to these phenomena. It has been reported that thrombocytosis becomes a risk factor to both the fetus and mother during pregnancy [28]. To explore this possibility, a peripheral blood assay was performed in non-pregnant mice using a Sysmex XT-2000iV hematology analyzer, because intracellular oxidative stress activates megakaryocytic maturation by Nrf-2 activation leading to increasing platelet number in peripheral blood [29]. An increased number of platelets (PLT-I and PLT-O), an abnormally large platelet ratio (P-LCR), and an increased platelet density (PCT) all characterized the peripheral blood of non-pregnant *Tet-mev-1*mice compared to wild-type C57BL/6j mice (Fig. 5A). In addition, the spleen weight, which is marker of inflammation (one risk factor for recurrent abortion), was dramatically increased in non-pregnant *Tet-mev-1*mice (Fig. 5B). These results suggest that the *mev-1* mutation (SDHC^V69E^) caused thrombocytosis and splenomegaly, contributing in a low delivery rate. These conditions were not changed in pregnant mice.

### Angiodysplasia with decreasing Flt-1 protein in placenta

**Fig. 6:**
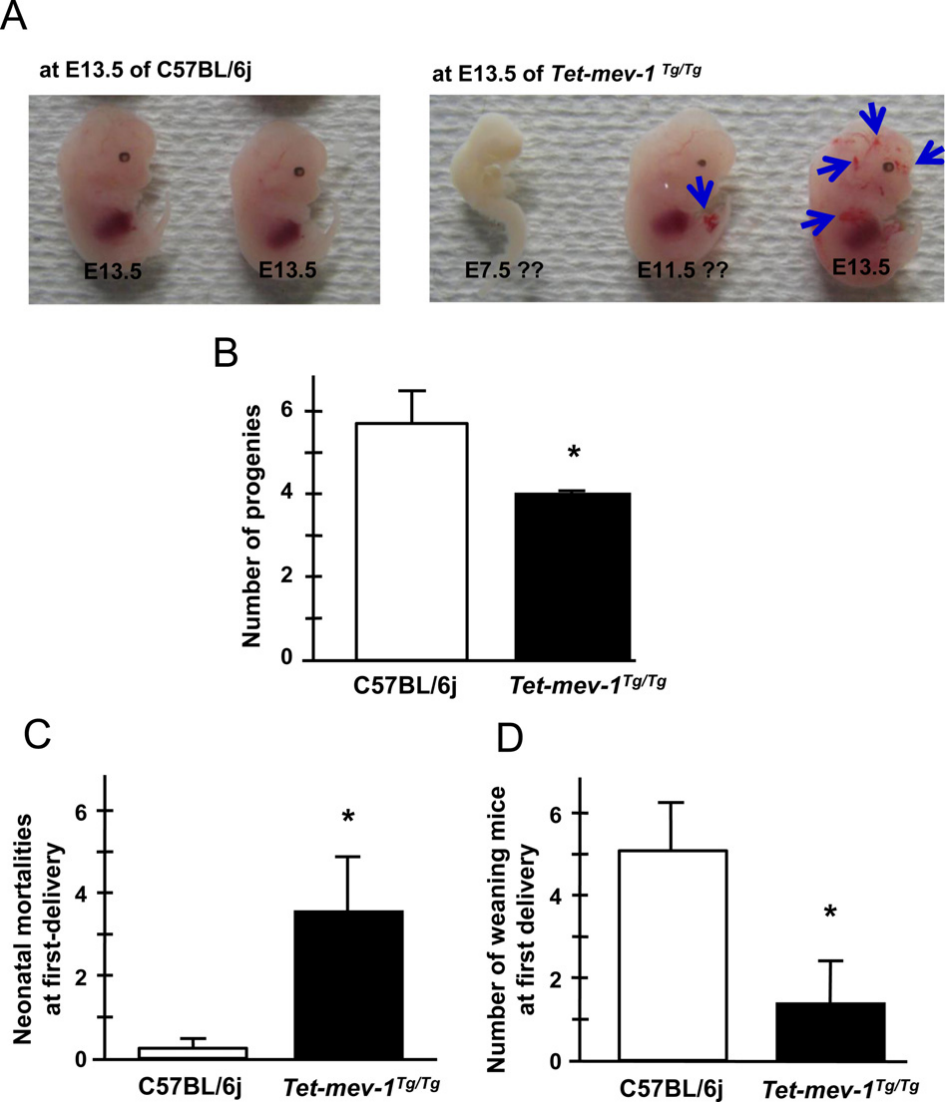
Neonatal mortalities as well as the number of progeny and weaning mice. (A) Mouse fetuses at 13.5-days of embryogenesis. The blue arrows indicate abnormal angiogenesis. (B) The number of progenies at first delivery. Results are expressed as mean ± SD; ^*^P = 0.01; *n* = 8 in C57BL/6j, *n* = 11 in *Tet-mev-1* mice. (C) Neonatal mortalities in the first delivery. Results are expressed as mean ± SD; ^*^P = 0.001; *n* = 8 in C57BL/6j, *n* = 11 in *Tet-mev-1* mice. (D) The number of weaning mice at first delivery. Results are expressed as mean ± SD; ^*^P = 0.001; *n* = 8 in C57BL/6j, *n* = 11 in *Tet-mev-1* mice.

Next, placentas were examined from pregnant mice. The vascular endothelial growth factors (VEGF) signaling is important in regulating vascular cell recruitment and proliferation for the placental formation [30,31]. It is well known that the most abundant and active member of the VEGF receptors is VEGF receptor-1 (VEGFR-1), also known as Flt-1. This binds to VEGF-A and PLGF-1 as a key factor promoting angiogenesis in the placenta. Disruptions contribute to the pathogenesis of female infertilities such as preeclampsia [32]. Flt-1 protein levels were dramatically decreased in morphologically normal placentas from 13.5-day pregnant *Tet-mev-1* mice, even though VEGF-A, estrogen receptor (ER)-a and ß protein levels were normal compared to control placentas of C57BL/6j mice (Figs. 5C–E and S1A and B). Additionally, the inflammatory cytokine levels, IFN?, IL-1ß and IL-6, were not changed in these placentas from 13.5-day pregnant *Tet-mev-1*mice (Fig. S1C–E). Given this, it is not surprising that developmental arrest and abnormal angiogenesis were observed at the 13.5-day-embryonic age in *Tet-mev-1*mice (Fig. 6A). As well, the number of progeny was significantly decreased in *Tet-mev-1* mice (Fig. 6B). The neonatal mortality was increased, thus the number of weaning mice was dramatically decreased at first delivery in *Tet-mev-1*mice (Fig. 6C,D). Surprisingly, the *Tet-mev-1* mice that successfully delivered, nurtured and weaned offspring had statistically normal numbers of progeny, neonatal mortality and weaning mice in ensuing pregnancies (Fig. S2A and B). Thus, the endogenous oxidative stress in these mice profoundly affected the first pregnancy but not subsequent ones.

## Discussion

We have demonstrated that a mitochondrial defect resulting from the SDHC^V69E^ mutation causes mitochondrial and intracellular oxidative stress in the nematode *C. elegans* *mev-1*[15], a *mev-1*-mimic transgenic *Drosophila* model [33] and mouse embryonic fibroblast SDHC E69 cells [18]. Recently, we have reported that *Tet-mev-1* mice expressing the SDHC^V69E^ transgene exhibit much the same effects [20]. The mutation site at the 69th position is located in a ubiquinone-binding region, changing the neutral and hydrophobic valine to an acidic and hydrophilic glutamate. This mutation causes decreased affinity between complex II and ubiquinone, leading to defects in electron transport. The leaked electrons react with nearby oxygen in mitochondria leading to mitochondrial and intracellular reactive oxygen species such as superoxide anion (O_2_^•-^), hydrogen peroxide (H_2_O_2_) and hydroxyl radical (^•^OH). The resultant intracellular oxidative stress causes excessive apoptosis during the mitotic cell divisions inherent to embryogenesis and neonatal development, resulting in low birth-weight infants and growth retardation in *Tet-mev-1* mice [20]. *Tet-mev-1* mice have normal food intake and water consumption. They recover body size and weight about 12 weeks after birth. Moreover, *Tet-mev-1* mice exhibit accelerated age-dependent corneal physiological and pathological phenotypes such as Fuchs's corneal dystrophy, keratoconus and dry eyes with keratitis and lacrimal gland inflammation, especially in male mice [23,34]. In this report, we have documented that mouse infertility also results from the *mev-1* mutation (SDHC^V69E^) and have identified its likely causation.

Male *Tet-mev-1* mice produce sperm in normal numbers and of wild-type morphology. This was initially surprising, given that mitochondrial-DNA deleted mouse models (mito-mice) result in male infertility owing to abnormal spermatogenesis and sperm morphology [9]. However, mito-mice likely have more severe phenotypes since the mitochondrial DNA deletions completely abolish the mitochondrial ATP production. In addition, these mice are reported to have mitochondrial structural defects that might impact spermatogenesis [9]. In contrast, the SDHC^V69E^ mutation coding transgene expression level has been tailored such that its expression equals that of endogenous wild-type SDHC protein. Consequently, *Tet-mev-1* mice are not influenced by the mitochondrial complex II weak dysfunctions during spermatogenesis except for sperm motility. The decreased mobility could be due to either changes in glucose and pyruvate metabolism or metabolic stores with chronic oxidative stress in the *Tet-mev-1* sperm.

Ovaries of *Tet-mev-1* mice showed considerable mitochondrial oxidative stress. They were swollen and vacuolated in ovarian interstitial cells. Moreover, the follicle maturations were not synchronously developed, leading to slightly decreasing ovulations in *Tet-mev-1* mice. In the *in vitro* fertilization assay, early embryogenesis through the blastocyst stage was completely normal; however, the fertilization rate was low, which was likely caused by seemingly healthy but defective unfertilized eggs and low sperm motility in *Tet-mev-1* mice. On the other hand, the fertilization rate was lower between cross-species gametes of C57BL/6j and *Tet-mev-1* conditional transgenic mice, respectively. This result likely reflects the fact that ATP synthesis is mainly through glycolysis in these blastomere developmental phases [27]. SDHC^V69E^ is one of complex II subunits, which works in conjunction with the citric cycle, and would therefore be expected to have minimal effects on early embryogenesis.

Non-pregnant *Tet-mev-1* mice exhibit an increased number of platelets (PLT-I and PLT-O), an abnormally large platelet ratio (P-LCR) and an increased platelet density (PCT). These thrombocytosis factors could contribute to decreased pregnancy and delivery rates. In addition, non-pregnant *Tet-mev-1* mice showed splenomegaly. This is consistent with the fact that 13.5-day pregnant *Tet-mev-1* mice had significant fetal loss and placental inflammation. In keeping with this, Flt-1 (VEGFR-1) was dramatically decreased in the morphologically-normal placentas of *Tet-mev-1* mice. These placental phenotypes lead to hypoxic conditions that partially explain the abnormal angiogenesis in embryos (Fig. 6A). On the other hand, VEGF-A, estrogen receptors a and ß, and inflammatory cytokine levels such as IFN?, IL-1ß and IL-6, which are also known as risk factors of contributing to abortion, were not changed in *Tet-mev-1* mice.

We have documented that female *Tet-mev-1* mice exhibit thrombocytosis, splenomegaly and placental angiodysplasia, which may constitute risk factors predisposing towards spontaneous abortion and recurrent miscarriage. In addition, fetal and neonatal *Tet-mev-1* mice frequently died by developmental arrest with an excessive apoptosis [20] and abnormal angiogenesis under the hypoxic condition through placental dysfunctions with decreased Flt-1 protein helps explain this. Interestingly, *Tet-mev-1* mice that were successful in one pregnancy had second deliveries at almost wild-type rates. This is worth exploring and is just one aspect that we feel makes *Tet-mev-1* mice a compelling animal model to study recurrent abortion.

Oxidative stress is present in most organs exposed to high oxygen metabolism such as the placenta. There is an emerging confluence of opinion suggesting that oxidative stress is one of the main underlying mechanisms in the pathogenesis of a continuum of disease processes such as spontaneous abortion and eclampsia. Oxidative stress and ROS-induced damage may be important missing pieces of the puzzle explaining abortions and recurrent pregnancy loss of unexplained etiology. Our study using *Tet-mev-1* mice clearly demonstrates that oxidative stress from mitochondria leads to spontaneous abortion and recurrent miscarriage with placental inflammation and embryonic developmental arrest resulting in fetal loss under the hypoxic conditions caused by abnormal angiogenesis in placenta. Human recurrent miscarriage in the United Kingdon is defined as three or more consecutive abortions during the first and second trimesters. A human abortion does not necessarily indicate a recurrent miscarriage is caused by maternal and fetal conditions in the first and second trimesters. *Tet-mev-1* mice have an embryonic arrest leading to fetal loss at early pregnancy and a placental inflammation at later-term pregnancy, thus mimicking the human conditions of miscarriage during the first or second trimesters. We have not yet clearly demonstrated why *Tet-mev-1* mice that experience a successful pregnancy are seemingly refractory to subsequent recurrent miscarriages, but we are actively exploring this interesting question. In conclusion, *Tet-mev-1* mice provide a powerful opportunity to study recurrent abortion with oxidative stress. Thus, reducing oxidative stress might be one possible treatment to combat spontaneous abortion and recurrent miscarriage.

## Materials and methods

### Animals

Wild-type C57BL/6j and *Tet-mev-1* conditional transgenic C57BL/6j background mice were treated with doxycycline hyclate (0.1 mg/ml doxycycline with 0.01 mg/ml saccharine or 2 mg/ml with 0.2% saccharine in drinking water) throughout prenatal development to adult. *Tet-mev-1* mice were constructed with the Tetracycline system that was uniquely developed by both rTetR (recombinant tetracycline receptor)-VP16 (reverse tetracycline-dependent transcriptional activator) and TetR-KRAB (tetracycline-dependent transcriptional repressor) as previously described [20]. This mouse model ubiquitously expresses the SDHC^V69E^ mutation-coding transgene in various tissues with doxycycline reagent to competitively and equally induce SDHC^V69E^ mutant protein to the endogenous SDHC protein level [18,20]. While suffering initial growth retardation, animals are of normal size after 12 weeks [20]. After 3 months, male and female *Tet-mev-1* mice show accelerated corneal dysfunctions with age, *i.e*., delayed epithelialization with keratitis, decreasing endothelial cells such as Fucks corneal dystrophy, and thickened Descemet's membrane [23]. Male *Tet-mev-1* mice also display lacrimal gland inflammation [34]. All animals were maintained on a 12 h light/dark cycle under an SPF condition: 22 ± 1 °C, approximately 40% humidity, 12/12-h light/dark cycle, a standard diet CE2 (9.3% water, 25.8% crude protein, 4.5% crude fat, 4.5% crude fiber, and 6.7% crude ash) (CLEA Japan, Inc.) and sterile water *ad libitum*. All protocols complied with the Guidelines for Animal Experimentation of Tokai University and were approved by the Tokai University Animal Care Committees.

### Mitochondrial reactive oxygen species measurement

The basal reactive oxygen species (ROS) levels in mitochondrial fraction proteins of tissues were measured in intact mitochondrial fraction proteins as previously described [20]. Basal ROS levels in mitochondrial fraction proteins were measured by the photon counter with an AB-2200 type Luminescencer-PSN (ATTO, Tokyo, Japan) at 37 °C using the chemiluminescent probe MPEC (2-methyl-6-*p*-methoxyphenylethynyl-imidazopyrazinone) (ATTO Co., Tokyo, Japan). The rates of ROS were expressed as counts per second and the amounts were calculated by subtracting the optical density of samples in the presence of 10 µg/ml bovine Cu, Zn-superoxide dismutase from that in the absence of the enzyme. Cu, Zn-superoxide dismutase was used as the compensation for only mitochondrial O_2_^•-^ level measurements in all cases.

### Carbonylated protein measurement

The mouse tissue protein extracts were prepared by the same protocol in western blot analysis. The DNPH-treated 200 ng proteins quantified protein carbonyls contents by enzyme-linked immunosorbent assay (ELISA) using 96-well plate (Corning Coster, Cambridge, MA). DNPH treatment methodology was as previously described [20]. The plates were incubated with anti-DNPH antibody in PBS containing 0.1% BSA, 0.1% Gelatin, 0.1% NaN_3_ , 1 mM MgCl_2_. After washing with PBS, the plates were incubated with HRP-conjugated rabbit IgG antibody, and then o-phenylenediamine (OPD) (Sigma Co., Tokyo, Japan) - H_2_O_2_ solutions were added. Finally, peroxidase activities were measured at 492 nm on SpectraMax 250 (Molecular Devices Co., Sunnyvale, CA) after 30-min incubation at room temperature followed by the addition of 1 M H_2_SO_4_.

### TUNEL staining

The segments of the tissues of 3 month-old mice were perfused and fixed with 4% paraformaldehyde in PBS under physiological pressure, and embedded in paraffin and sectioned at a thickness of 2–3 µm. TUNEL reactions were performed using TUNEL Enzyme, TUNEL Dilution Buffer and Biotin-16-dUTP (Roche Diagnostics Corp., Tokyo, Japan) according to the manufacturer's instructions. Sections were then incubated with anti-streptAvidin-HRP conjugated antibody (1:40 dilution), developed using diaminobenzidine [20 mg DAB, 65 mg NaN_3_, 17 µl H_2_O_2_ in 100 ml 0.05 M Tris–HCl buffer], and treated in methyl green nucleus staining.

### Total sperm number, sperm motility, and *in vitro* fertilization assays

Sperm were collected from the incubated cauda epididymidis in 1 ml of HTF medium, and the total sperm number was counted. These sperm samples were also used for the motility and *in vitro* fertilization assays. After 2 h incubation in HTF medium, the activated sperm could swim up to the upper layer of the medium within the incubation time, the swim-up sperm were counted as the number of motile sperm in 1.5 ml HTF medium after 2, 4 and 6 h of incubation. Female mice were induced to superovulate by consecutive injections of pregnant mare serum gonadotropin and human chorionic gonadotropin (hCG) with an interval of 48 h between injections. Unfertilized oocytes were collected from the oviducts 15 h after the hCG injection. The *in vitro* fertilization was carried out by using activated sperm collected from cauda epididymidis in HTF medium. The developmental frequency of embryos was measured in KSOM medium in a 37 °C incubator.

### Western blot analysis

Forty micrograms of total protein extract denaturalized by boiling after the addition of 2 × SDS-PAGE sample buffer [0.125 M Tris–HCl (pH 6.8), 10% 2-mercaptoethanol, 4% SDS, 10% sucrose and 0.004% bromophenol blue]/lane were resolved using 10–20% SDS-PAGE gradient gel and analyzed by Western blots. After electrophoresis, the proteins were transferred to PVDF (polyvinylidene difluoride) membrane Clearblot membrane (ATTO) using a Semi-dry blotting machine AE-6677 (ATTO). To block nonspecific protein binding, membranes were treated for 2–8 h at 20–25 °C with 5% bovine serum albumin (EQUITECH-BIO, Inc: #BAH64), 0.1% Tween-20 in TBS [0.02 M TRIZMA BASE, 0.137 M NaCl (pH 7.6)]. Primary antibodies [Rabbit monoclonal [Y103] to VEGF Receptor 1 (abcam: ab32152), Rabbit anti-mouse Vascular Endothelial Growth Factor (Millipore: ab1876), Rabbit monoclonal antibody [E115] to Estrogen Receptor alpha (GeneTex: GTX61047), Rabbit polyclonal Estrogen receptor beta antibody (abcam: ab3576), Rat monoclonal [RMMG-1] to Interferon gamma antibody (abcam: ab24979), Rabbit polyclonal to IL-1 beta antibody (abcam: ab9722), Rabbit polyclonal to IL-6 antibody (abcam: ab6672), Goat polyclonal to beta Actin antibody (abcam: ab8229), and Rabbit polyclonal antibody to Beta-Actin (GeneTex: GTX110564)] were used in TBS containing 5% bovine serum albumin in each condition. Actin was used as an internal control protein for loading normalization of the quantification analysis. Horseradish peroxidase-coupled specific secondary antibodies [anti-rabbit IgG, HRP-linked antibody (Cell Signaling Technology: #7074) or polyclonal rabbit anti-goat immunoglobulines/HRP antibody (Dako: P0449)] were incubated in TBS containing 5% bovine serum albumin for 2 h at room temperature in each condition. The detection system used was ECL-plus Western Blotting Detection Reagent (GE Healthcare, UK). The chemiluminescence signals were visualized under LAS3000 mini (Fuji Photo Film Co., Japan). Quantitative densitometric analysis was done using Multi Gauge Ver3.0 (Fuji Photo Film Co., Japan).

### Data analyses

Statistically measurements were analyzed by one-way ANOVA, Bonferroni post hoc comparisons and a two-tailed, unpaired student's *t*-test. All values in the figures and figure legends are means ± SD.

## Appendix A Supplementary Materials

Supplementary Materials for Genetically induced oxidative stress in mice causes thrombocytosis, splenomegaly and placental angiodysplasia that leads to recurrent abortion.
