# Supplementary material for: Genetically induced oxidative stress in mice causes thrombocytosis, splenomegaly and placental angiodysplasia that leads to recurrent abortion
Source: Redox Biol. 2014 May 14;2:679–85. doi: 10.1016/j.redox.2014.05.001 (PMC4052530; doi:10.1016/j.redox.2014.05.001)
Supplement: Supplementary file 1 — Supplementary Materials for Genetically induced oxidative stress in mice causes thrombocytosis, splenomegaly and placental angiodysplasia that leads to recurrent abortion. [file mmc1.docx]

**
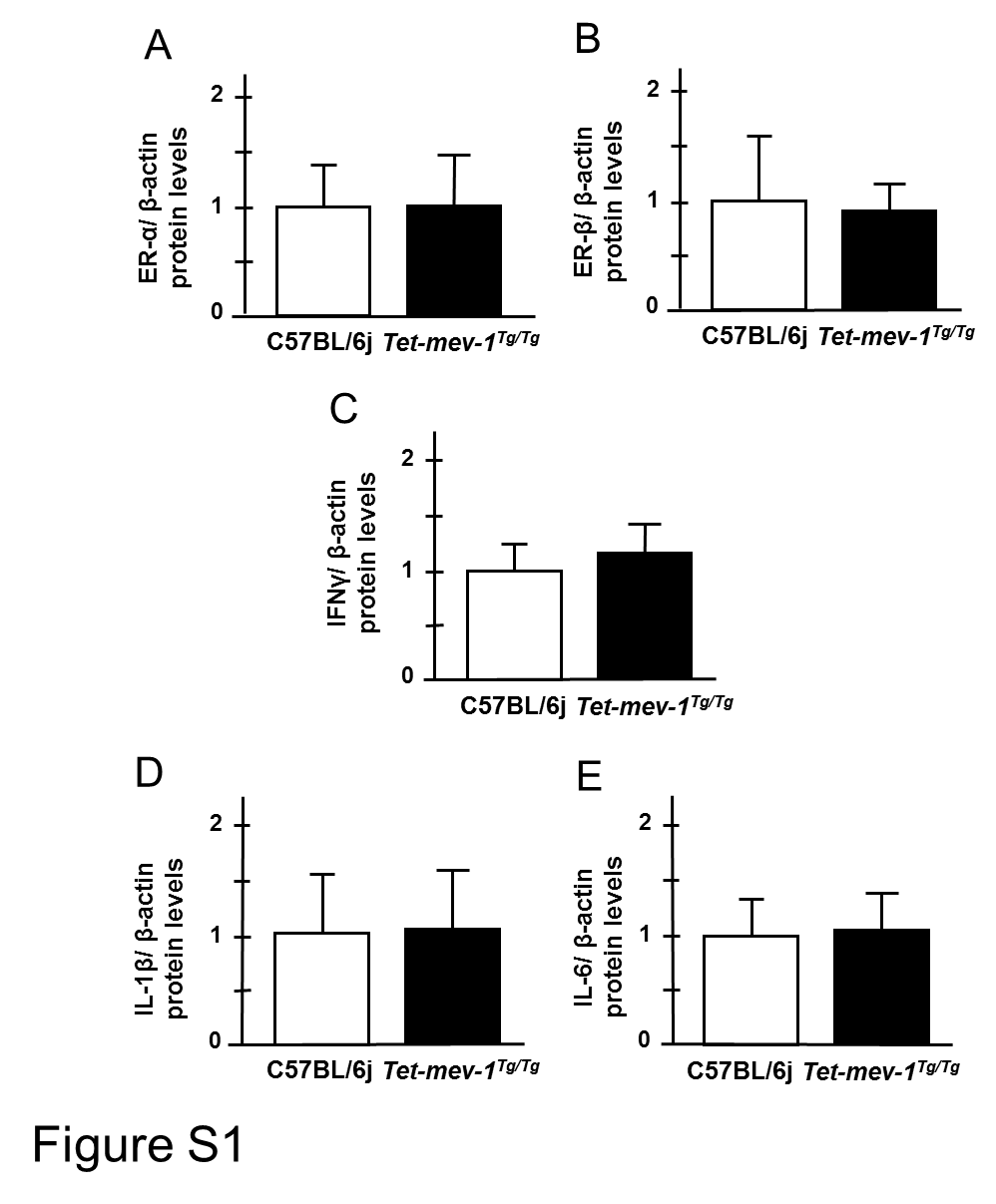
**

**Fig. S1.**  The results of western blot analysis. (*A*) The Estrogen receptor (ER)-α protein levels by western blot analysis using Rabbit monoclonal antibody [E115] to Estrogen Receptor alpha (GeneTex: GTX61047) to probe normal placenta of pregnant wild-type C57BL/6j and *Tet-mev-1* mice. (*B*) The Estrogen receptor (ER)-β protein levels by western blot analysis using Rabbit polyclonal Estrogen receptor beta antibody (abcam: ab3576) to probe normal placenta of pregnant wild-type C57BL/6j and *Tet-mev-1* mice. (*C*-*E*) The cytokines protein levels by western blot analysis using Rat monoclonal [RMMG-1] to Interferon gamma antibody (abcam: ab24979) (*C*), Rabbit polyclonal to IL-1 beta antibody (abcam: ab9722) (*D*), Rabbit polyclonal to IL-6 antibody (abcam: ab6672) (*E*) to probe normal placenta of pregnant wild-type C57BL/6j and *Tet-mev-1* mice. All data are expressed as mean ± SD; *n* = 5 in each group.

**
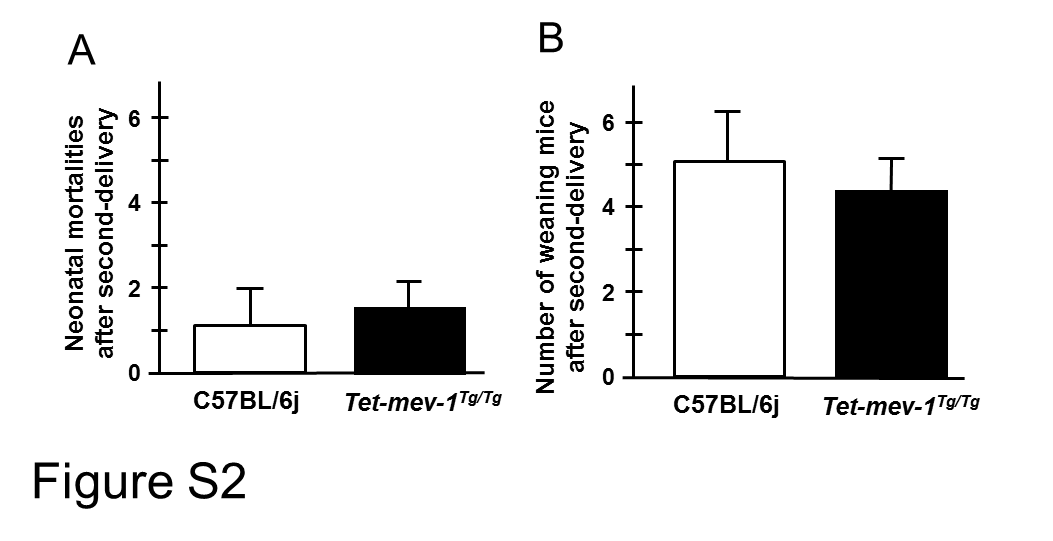
**

**Fig. S2.** Neonatal mortalities and the number of weaning mice at second delivery after one successful delivery. (*A*) Neonatal mortality at second delivery after one successful delivery. Results are expressed as mean ± SD; *n* = 8 in C57BL/6j, *n* = 11 in *Tet-mev-1* mice. (*B*) The number of weaning mice at second delivery after one successful delivery. Results are expressed as mean ± SD; *n* = 8 in C57BL/6j, *n* = 11 in *Tet-mev-1* mice.
